# Linking the unfolded protein response to bioactive lipid metabolism and signalling in the cell non‐autonomous extracellular communication of ER stress

**DOI:** 10.1002/bies.202300029

**Published:** 2023-05-15

**Authors:** Nicole T. Watt, Anna McGrane, Lee D. Roberts

**Affiliations:** ^1^ Leeds Institute of Cardiovascular and Metabolic Medicine University of Leeds Leeds UK

**Keywords:** ceramide, endoplasmic reticulum, lipidomics, lipids, paracrine signalling, skeletal muscle, unfolded protein response

## Abstract

The endoplasmic reticulum (ER) organelle is the key intracellular site of both protein and lipid biosynthesis. ER dysfunction, termed ER stress, can result in protein accretion within the ER and cell death; a pathophysiological process contributing to a range of metabolic diseases and cancers. ER stress leads to the activation of a protective signalling cascade termed the Unfolded Protein Response (UPR). However, chronic UPR activation can ultimately result in cellular apoptosis. Emerging evidence suggests that cells undergoing ER stress and UPR activation can release extracellular signals that can propagate UPR activation to target tissues in a cell non‐autonomous signalling mechanism. Separately, studies have determined that the UPR plays a key regulatory role in the biosynthesis of bioactive signalling lipids including sphingolipids and ceramides. Here we weigh the evidence to combine these concepts and propose that during ER stress, UPR activation drives the biosynthesis of ceramide lipids, which are exported and function as cell non‐autonomous signals to propagate UPR activation in target cells and tissues.

## Graphical abstract

The endoplasmic reticulum (ER) is key to cellular protein and lipid biosynthesis. ER stress, in response to lipotoxicity in metabolic diseases, initiates the Unfolded Protein Response (UPR), reducing ER protein load. We discuss emerging evidence that the UPR regulates cell non‐autonomous bioactive lipid signals that propagate systemic ER stress.

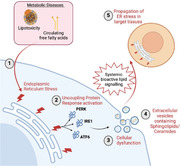

**Abbreviations**
ATF6: activating transcription factor 6
CHOP: CCAAT/enhancer binding protein (C/EBP) homologous protein
elF2α: eukaryotic translation initiation factor 2α
ER: endoplasmic reticulum
IRE1: inositol‐requiring enzyme 1
PERK: protein kinase r‐like endoplasmic reticulum kinase
T2DM: type 2 diabetes mellitus
UPR: unfolded protein response
XBP‐1: X box‐binding protein‐1

## INTRODUCTION

The Endoplasmic Reticulum (ER) is a cellular organelle crucial for protein synthesis and folding, lipid biosynthesis and calcium homeostasis. Disrupted ER function results in organelle stress and misfolded protein accumulation. Unchecked unfolded protein accretion can result in cell death. ER stress contributes to the pathophysiology of many diseases including ageing, tumorigenesis and metabolic diseases such as obesity and type 2 diabetes mellitus (T2DM).^[^ ^1^, ^2^ ^]^ An increase in circulating saturated fatty acids, such as palmitate, characterise metabolic diseases including obesity, T2DM and dyslipidemia.^[^ ^3^, ^4^ ^]^ These elevated fatty acids promote metabolic dysfunction through a process called ‘lipotoxicity’ in insulin‐responsive tissues. ER stress has emerged as a potential unifying mechanism linking lipotoxicity to metabolic dysfunction and disease.^[^ ^5^, ^6^, ^7^ ^]^ However, the mechanisms linking lipotoxicity with the induction of ER stress remain to be fully elucidated.

The cell has protective adaptations to maintain protein homeostasis and cell survival during ER stress. ER stress results in the activation of the unfolded protein response (UPR), a protective signalling cascade consisting of three arms, mediated by the kinases protein kinase R‐like endoplasmic reticulum kinase (PERK) and inositol‐requiring enzyme 1 (IRE1), and the transcription factor, activating transcription factor 6 (ATF6), respectively. Signalling through these proteins increases protein chaperones and disulphide isomerases, activates protein degradation and inhibits protein translation.^[8]^ These responses reduce ER protein load. However, chronic UPR activation can lead to cell death. The intracellular mechanisms of ER stress and UPR regulation are relatively well understood.

Emerging evidence suggests that UPR activation can be propagated in a paracrine and systemic manner through cell non‐autonomous signalling mechanisms.^[^ ^9^, ^10^ ^]^ The nature of these signals remains poorly understood. Based on our recent publication exploring lipotoxicity‐induced UPR regulation of bioactive lipids,^[11]^ we hypothesise that during ER stress the unfolded protein response directly regulates the synthesis of integrative bioactive lipid signals that, when secreted, propagate ER stress and UPR activation through a cell non‐autonomous mechanism.

Here, we will briefly introduce the ER and the key molecules regulating, and participating in, the UPR. We will outline the emerging evidence for cell non‐autonomous extracellular and interorgan communication as a means of transmitting systemic ER stress. We will then discuss the relationship between UPR activation and sphingolipid metabolism that suggests a role for bioactive lipids as integrative signals of ER stress. Finally, we will introduce and evaluate the concept of cell non‐autonomous paracrine/endocrine signalling by bioactive lipids during ER stress.

## ENDOPLASMIC RETICULUM STRESS AND THE UPR

The ER is a large, dynamic intracellular membrane system that plays an essential role in protein synthesis, folding and transport.^[2]^ It is crucial for proteostasis within the cell, ensuring that the entire life sequence of a protein from its biogenesis, through folding and assembly, trafficking and final degradation proceeds efficiently.^[^ ^2^, ^12^ ^]^ The dynamic nature of cellular homeostasis means that the flux of proteins through the ER is highly variable depending on cellular demand. Numerous control mechanisms are in place to ensure that anterograde protein transport is maintained. However, if there is an imbalance between proteins entering the ER and the capacity of the ER machinery to bring about efficient protein folding, there is a failure of ER homeostasis, and un‐ or mis‐folded protein chains accumulate in the ER. In this scenario, the organelle is regarded as under ‘stress’ which, if not resolved, is lethal to the cell.

**FIGURE 1:**
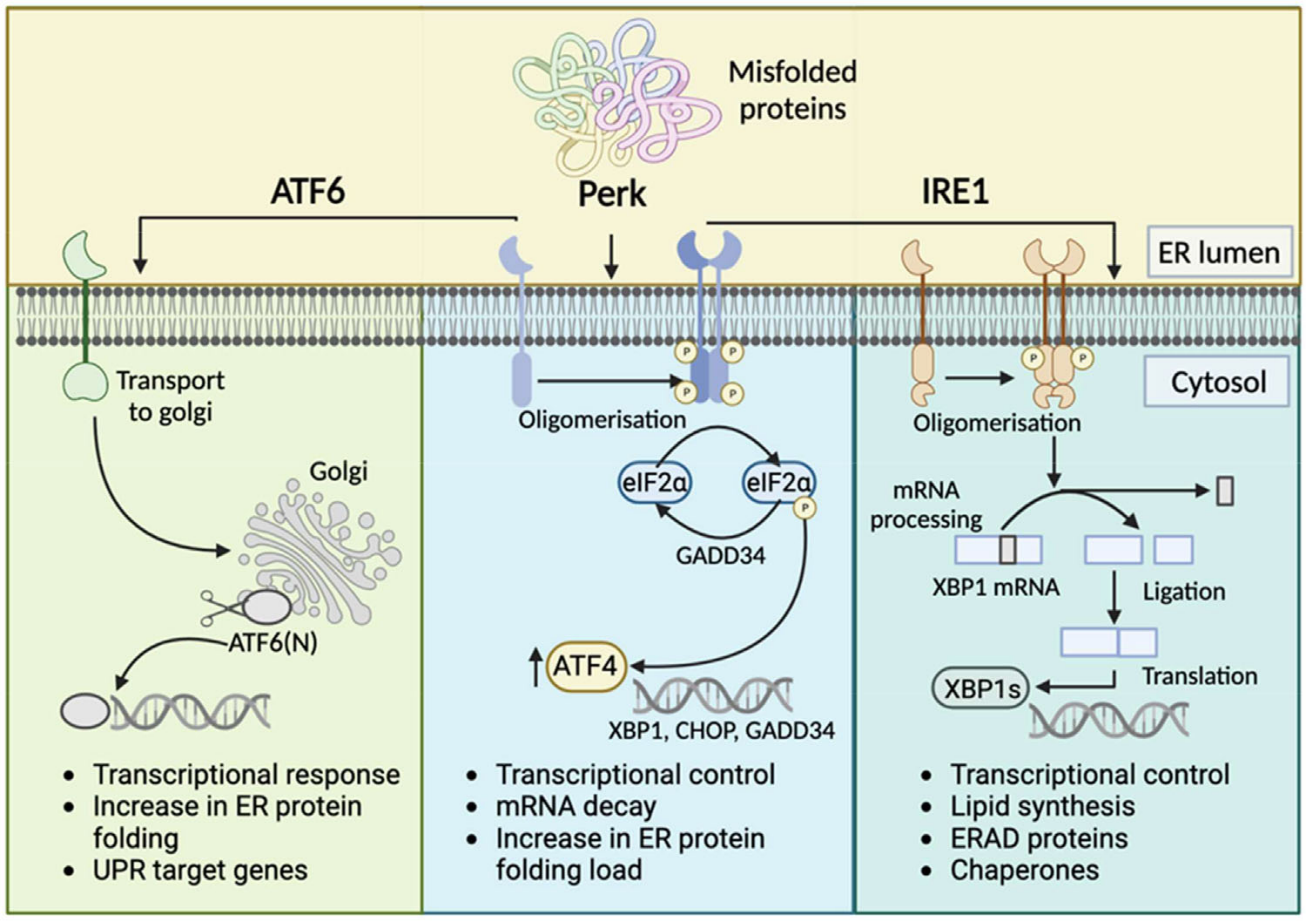
Schematic representation of the three branches of the endoplasmic reticulum (ER) unfolded protein response (UPR). In the ATF6 pathway, proteins are packaged into vesicles and delivered to the golgi where the luminal domain is removed by site‐1 and 2 proteases. ATF6(N) can then move into the nucleus to activate expression of the UPR target genes encoding BiP and GRP94. PERK oligomerisation and phosphorylation of itself and eIF2α transcription factor reduces protein flux through the ER. Reduced eIF2α increases the translation of ATF4. ATF4 target genes include those encoding UPR proteins CHOP, GADD34 and XBP1. Activated IRE1 by oligomerisation results in the cleavage of XBP1 mRNA to produce spliced, activated XBP1 (XBP1s). XBP1s regulates lipid biosynthesis and ER‐associated degradation components. ATF6 (activating transcription factor 6), ATF6(N) (N terminal cytosolic fragment of ATF6), PERK (protein kinase R‐like ER kinase), eIF2 (eukaryotic initiation factor 2), GADD34 (growth arrest and DNA damage‐ inducer 34), XBP1 (X box binding protein 1), CHOP (transcription factor C/EBP homologous protein), IRE1 (inositol‐requiring enzyme 1), XBP1s (activated XBP1), ERAD (endoplasmic reticulum associated degradation) (Created with BioRender.com).

The build‐up of un‐ or mis‐folded proteins in the ER lumen triggers a subset of ER membrane resident proteins to activate the UPR. The UPR is a collection of three signalling cascades that run in parallel with significant cross‐talk; protein kinase R‐like ER kinase (PERK)‐eukaryotic translation initiation factor 2α (elF2α), inositol‐requiring enzyme 1 (IRE1 α and β)‐X‐box binding protein 1 (XBP‐1) and activating transcription factor 6 (AFTK6 α and β) (see Figure 1).^[2]^ Once activated, the purpose of the UPR is to restore correct protein folding in the ER and thus prevent cytotoxicity.

Activation of PERK phosphorylates elF2α, which transiently stops the induction of mRNA translation but also upregulates the translation of specific UPR‐related genes including ATF4. Once translated, ATF4 translocates to the nucleus to further activate UPR genes that encode proteins in the antioxidant response and in amino acid biosynthesis and transport.^[2]^ ATF4 also transactivates CCAAT/enhancer binding protein (C/EBP) homologous protein (CHOP) which forms heterodimers with ATF4 to further advance UPR signalling, autophagy and mRNA translation. Dephosphorylation of elF2α occurs once normal protein folding has been restored which allows mRNA translation to reinitiate.^[2]^ Therefore, PERK activity functions to reduce total protein synthesis and protein translocation to the ER.

Alternatively, IRE1α‐simulated endonuclease activity enables the spliced form of XBP‐1 (XBP1s) to enter the nucleus and promote the upregulation of genes that enhance the capacity of the cell for protein folding, transport and degradation. IRE1α also mediates IRE1‐dependent decay (a form of regulated mRNA and microRNA decay) to moderate the protein‐folding demand on the ER.^[^ ^2^, ^13^, ^14^, ^15^ ^]^ ATF6α acts to modify the adaptive response to ER protein misfolding by increasing the availability of protein‐folding chaperones as well as increasing cellular protein degradation.^[2]^ By decreasing protein synthesis and translocation to the ER, whilst concomitantly increasing the resident protein folding machinery, the UPR attempts to restore normal cellular proteostasis.

It is worthy of note that whilst the balance between demand and capacity for protein synthesis can lead to an accumulation of un‐ or mis‐folded proteins, a disruption in intracellular calcium homeostasis can also result in ER stress through UPR‐independent mechanisms. Calcium ion concentrations are tightly regulated within cells and are sequestered within membrane‐bound organelles (including the ER and lysosomes) to prevent deleterious and unregulated effects. Sarco‐/endoplasmic reticulum calcium ATPase (SERCA) pumps reside in the ER membrane to maintain the high internal calcium concentrations required by the ER‐resident chaperones and post‐translational modification enzymes to support appropriate protein synthesis and folding.^[14]^ Following calcium dyshomeostasis, chaperone and enzymatic activity in the ER is reduced resulting in the initiation of ER stress. There is increasing interest in the interaction between lipotoxicity‐inducing free fatty acids such as palmitate and ER calcium handling in the initiation of UPR‐independent ER stress. Lysosomal calcium release has recently been implicated in a mechanism of hepatic lipotoxicity induced by palmitic acid.^[17]^ Evidence from β‐cells also suggests that palmitic acid depletes ER calcium stores resulting in ER stress.^[18]^ In addition, increased extracellular palmitate concentrations impair the ER's ability to maintain calcium stores in hepatocytes, resulting in ER stress‐mediated cellular dysfunction.^[19]^ A role for lipid—ER stress calcium homeostasis interactions in the induction of UPR‐independent ER stress has been reviewed elsewhere.^[20]^ This review will focus on the significance of ER stress‐induced, UPR‐mediated extracellular lipid signalling.

It is well established that misfolded proteins within a cell can induce ER stress. The contribution of cellular ER stress to disease progression, however, is less clear. The most likely scenario is that rather than the disease being caused by a loss of function from the correctly‐folded protein, the misfolded protein has a dominant, but detrimental, effect on cellular activity which results in cell dysfunction.^[12]^ However, if the combined effects of reduced protein synthesis and increased protein folding capacity are insufficient to restore ER proteostasis, prolonged UPR activation can become maladaptive and apoptosis is initiated. Although a less well‐understood component of the UPR, activation of apoptosis may be necessary to protect tissues or organisms from the detrimental effects of a cell containing an accretion of harmful, misfolded proteins.^[21]^

The ER stress‐induced cell death response involves a number of molecules also involved in the canonical UPR. IRE1α has been described as the rheostat capable of influencing the fate of the ER‐stressed cell.^[22]^ Binding immunoglobulin Protein (BiP) desensitizes IRE1α to low levels of stress and modulates the speed at which IRE1α deactivation occurs once ER stress has been alleviated. The downstream signalling from IRE1α, therefore, mediates the result of ER stress in the cell and determines cell survival.^[23]^ Recruitment of tumour necrosis factor (TNF) receptor‐associated factor 2 (TRAF2) to activated IRE1α in turn activates c‐Jun N‐terminal kinase (JNK) to cause apoptosis. PERK activation can also result in apoptosis through the PERK‐elF2α‐ATF4‐CHOP pathway.^[22]^ CHOP induces the expression of numerous pro‐apoptotic genes, activates ER oxidase 1α to increase the production of reactive oxygen species (ROS) and Ca^2+^‐efflux from the ER, and mRNA translation is restored through the formation of CHOP‐ATF4 heterodimers.^[22]^ Mitochondria take up the Ca^2+^ released from the ER to generate additional ROS. Cell death ensues due to cellular oxidative stress and impaired mitochondrial function in a CHOP‐dependent mechanism.^[22]^ Similarly, Bax/Bcl‐2 family members can increase ER permeability, particularly to calcium, to induce apoptosis.^[22]^

Recent attention has focussed on understanding the factors regulating ER stress‐induced apoptosis. This has been especially prominent in cancer research where regulation of ER stress‐induced apoptosis has been proposed to mediate tumour progression^[24]^ and drug resistance in solid tumours.^[25]^ Tumorigenesis may ensue when a dysfunctional cell under ER stress, which should be selectively removed through apoptosis, remains in the population. Most recently, a role for ferroptosis, a novel form of iron‐dependent programmed cell death, has been implicated in the response to fatal ER stress.^[26]^ However, questions remain as identification of the pathways mediating lethal ER stress (i.e., apoptosis competent) are currently understood to be the same as those mediating sub‐lethal ER stress (i.e., apoptosis incompetent). This suggests that it must be the duration or magnitude of the stress,^[22]^ or the genetic background, that must influence the cellular fate downstream of ER stress signalling pathway activation.

**FIGURE 2:**
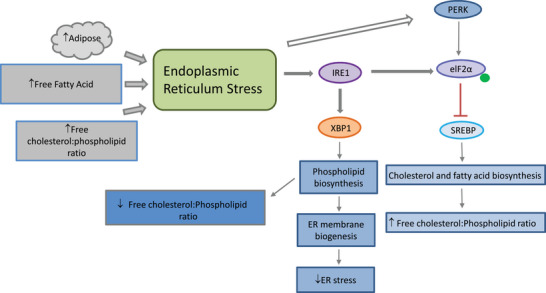
ER stress, induction of the unfolded protein response and its implications for cellular lipid metabolism. Increases in the ratio of free cholesterol:phospholipid, such as that seen in obesity, can induce ER stress and induce the unfolded protein response. Activation of IRE1 increases XBP‐1‐dependent phospholipid biosynthesis and ER membrane biogenesis to reduce the ratio of cholesterol:phospholipid and alleviate ER stress. Protein kinase RNA (PKR)‐like ER kinase (PERK)‐dependent eukaryotic translation initiation factor 2α (elF2α) phosphorylation opposes sterol‐regulated enhancer binding protein (SREBP) activation thereby reducing cholesterol synthesis and preventing further increases in cholesterol concentration.

Whilst the role of the UPR appears to be to enable a cell to respond robustly to the proteostatic demands being placed upon it rather than to specifically protect against ER stress, the UPR also has a role in the maintenance of the cellular lipidome. The ER is the main intracellular site for the biosynthesis of lipids and steroids including cholesterol, glycerophospholipids, sphingolipids and ceramides.^[27]^ The UPR also contributes to the expansion of membrane lipids in cells with a high secretory burden. Increases in membrane cholesterol, such as that seen in metabolic disease and obesity, can cause ER stress due to a perturbation of the inherent cholesterol:phospholipid ratio (see Figure 2). Activation of XBP‐1 activity increases the biosynthesis of phospholipids and enhances membrane biogenesis thus reducing the cholesterol:phospholipid ratio.^[28]^ Elf2α‐mediated attenuation of sterol‐regulated enhancer binding protein (SREBP) activity is also thought to further reduce cholesterol synthesis, though the mechanism is still unclear.^[29]^ This suggests that the UPR and SREBP‐activated signalling pathways work together to maintain lipid homeostasis.

We propose that the induction of ER stress and the activation of the UPR links directly to the regulation of sphingolipid metabolism and the production of bioactive lipid signals that contribute to the cell non‐autonomous propagation of systemic ER stress. We also suggest that by defining the mechanisms of ER‐stress activated bioactive lipid production, packaging, secretion, and UPR activation, we may uncover therapeutic targets for lipotoxicity‐associated metabolic diseases.

## THE CELL NON‐AUTONOMOUS EXTRACELLULAR AND INTERORGAN COMMUNICATION OF ER STRESS

Historically, the UPR was considered a signalling mechanism designed to respond to misfolded proteins wholly within the cell. However, recent developments have suggested that organisms have the ability to coordinate a systemic response to ER stress through communication to distal tissues.^[^ ^9^, ^10^, ^30^ ^]^ This occurs by communicating UPR activation between tissues, in a tissue‐specific, cell non‐autonomous endocrine manner via the release of extracellular signalling molecules. It has been proposed that this response allows cell types that are more susceptible to ER damage to prepare for ER stress, via the activation of the UPR in these tissues.^[30]^

There is increasing experimental evidence that cell non‐autonomous signalling regulates the UPR and ER stress. An example is provided by a study investigating the role of the IRE1 pathway in age‐onset loss of UPR function. Many cellular processes decline with age, one of which is the attenuation of the UPR. Taylor and Dillin hypothesised that the induction of the UPR may reverse the loss of ER proteostasis associated with ageing.^[30]^ The researchers found that the prolonged expression of the IRE1 pathway protein XBP1s in neuronal tissue of C. *elegans* was sufficient to induce UPR chaperone expression in distal tissues including the intestine. This process rescued age‐onset UPR resistance. Neuronal‐specific XBP1s expression also extended the C. *elegans* lifespan. This specific, one‐directional, non‐autonomous signalling was dependent on unc‐13, a mediator of small clear vesicle (SCV) release in neurons.^[31]^ This suggested that it was the release of neurotransmitters from SCV that was necessary for the propagation of the signal from the neuronal tissue to the intestine. Experiments in mice provide further evidence for an XBP1s‐mediated mechanism for cell non‐autonomous UPR activation. XBP1s was specifically expressed in the proopiomelanocortin neurons in the hypothalamus of mice. This tissue‐specific UPR activation leads to the activation of the UPR in hepatocytes and adipocytes and contributed to changes in the systemic metabolic physiology of the mice to counter diet‐induced obesity.^[32]^

Cell non‐autonomous UPR signalling may also contribute to the systemic regulation of immunity and the response to pathogens. Octopamine G protein‐coupled receptor (OCTR‐1), a putative octopamine G protein‐coupled catecholamine receptor, when expressed in specific sensory neurons of C. *elegans*, induced the expression of UPR and UPR target genes in distal immune tissues including phagocytes and the cells of immune barriers including the pharynx and intestines.^[^ ^33^, ^34^ ^]^ In an additional example of endocrine signalling in the induction of the UPR, regulating the immune response, both prostate and mammary cancer cells treated with UPR‐inducing agents produce unknown signals that activate the UPR in macrophages.^[^ ^9^, ^35^ ^]^ This process may link UPR‐mediated endocrine signalling with tumourigenesis.

Together these studies provide evidence that the tissue‐specific cell non‐autonomous activation of an ER stress response can coordinate UPR activation in distal tissues through systemic signalling mechanisms. Intriguingly multiple tissue and stress stimuli‐specific cell non‐autonomous signals operating through autocrine, paracrine and endocrine mechanisms are exhibited. This systemic coordination may facilitate a whole‐body response to levels of elevated stress represented by adverse environmental conditions or disease.

## THE UPR REGULATES SPHINGOLIPID METABOLISM AS AN INTEGRATIVE SIGNAL OF ER STRESS

**FIGURE 3:**
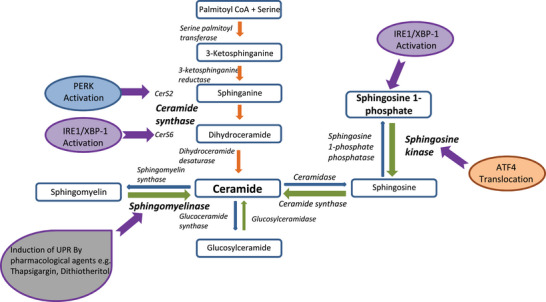
Schematic representation of the interaction between the Unfolded Protein Response (UPR) and the ceramide synthesis pathway. The biosynthetic pathway of ceramide synthesis is known to be directly influenced by pharmacological agents or oxidative stressers which induce the UPR. Pathways which show positive upregulation are shown by thickened arrows and specific targets are shown in bold typeface. PERK^[^ ^11^, ^47^ ^]^ and IRE1/XBP1^[^ ^45^, ^48^ ^]^ activation by the UPR have been identified as directly upregulating ceramide synthase in the de‐novo synthesis pathway. However, IRE1 and ATF4 can also act through the sphingosine kinase‐mediated ceramide salvage pathway.^[^ ^46^ ^]^

As mentioned previously, the ER is a key site of not only protein synthesis, but also lipid metabolism. Although lipids have been traditionally considered as inert molecules providing the primary components of cell membranes or as efficient energy stores and fuels, they are now understood to also function as signalling mediators in intracellular, extracellular and endocrine processes. These signalling lipids are termed ‘bioactive lipids’ and contribute to diverse homeostatic functions including immunity, inflammation, tissue homeostasis^[^ ^36^, ^37^ ^]^ and the regulation of whole‐body energy metabolism.^[^ ^38^, ^39^ ^]^ Bioactive lipids are divided into four groups based on their biochemical classification; eicosanoids, specialised pro‐resolving mediators, endocannabinoids and of particular relevance to ER stress, lysoglycerophospholipids/sphingolipids/ceramides. The ceramide lipid class consists of a sphingoid long chain base attached to a fatty acid by an amide bond. De novo ceramide synthesis occurs in the ER through a metabolic pathway beginning with L‐serine and palmitoyl‐CoA condensation by serine palmitoyl transferase.^[40]^ This produces 3‐ketosphinganine, which is reduced to sphinganine by the enzyme 3‐ketosphinganine reductase.^[41]^ Sphinganine is acetylated by ceramide synthases (CerS) to dihydroceramide. CerS are a family of six enzymes (CerS 1–6) each with a distinct preference for varying acyl chain lengths. The enzyme dihydroceramide desaturase catalyses the final step, the reduction of dihydroceramide to ceramide (see Figure 3). Given the ERs key role in ceramide synthesis, integrating signalling via bioactive ceramide lipids to communicate UPR status appears advantageous.

Recent studies suggest the UPR can regulate sphingolipid and ceramide metabolism. The induction of the UPR, using the pharmaceutical compound thapsigargin, in pancreatic β‐cell‐derived insulinoma cells induced the expression of the ceramide synthetic enzyme neutral sphingomyelinase,^[^ ^42^ ^]^ and increased intracellular ceramide concentrations resulting in apoptosis.^[^ ^42^ ^]^ In a corroboratory study in insulinoma cells, induction of the UPR using dithiothreitol (DTT) also increased the cellular ceramide concentration through an increase in CerS6 expression.^[43]^ Tam et al. exposed HEK293 cells to both thapsigargin and DTT to induce ER stress and also observed an increase in dihydroceramide and dihydrosphingosine cellular concentrations.^[44]^

Specific regulation of sphingolipid and ceramide synthesis by the UPR appear to be distinguished by differential effects mediated by the three UPR pathways (IRE1, ATF6 and PERK). Park et al. induced ER stress in human keratinocytes using pharmacological agents and observed an increase in cellular ceramides, sphingosine and sphingosine‐1‐phosphate (S1P). S1P increased the innate immune regulator cathelicidin antimicrobial peptide (CAMP). IRE1 also regulates CAMP expression, suggesting that sphingolipids may be both regulated by the UPR, and function as mediators of UPR activation.^[45]^

The regulation of sphingolipid metabolism by the UPR translates in vivo, with an ATF4 signalling pathway implicated. Expression of the ceramide synthesis genes sphingosine kinase 2, CerS3 and acid ceramidases 2 and 3 was increased in the liver of mice treated with pharmacological UPR inducer tunicamycin, through an ATF4‐mediated mechanism.^[46]^

The UPR kinase PERK has also been implicated in the regulation of ceramide metabolism. Expression of the cytokine Interleukin 24 (IL24) led to PERK activation and increased ceramide concentrations in glioblastoma cells.^[47]^ This process drove apoptosis of the cells. Transgenic inhibition of PERK abrogated both the increase in glioblastoma cell ceramides and apoptosis initiated by IL24. This suggested that the ceramides may have contributed to the pathways of PERK‐mediated apoptosis.

As discussed above, a number of studies have provided associative evidence that changes in lipid metabolism occur as a result of an ER stress‐induced, UPR mediated mechanism.^[^ ^31^, ^32^, ^33^, ^34^, ^35^ ^]^ A key question remained as to whether the UPR directly mediates lipid metabolism and whether this response is integrated as part of downstream cellular and systemic responses to ER stress. We will now highlight recent discoveries that begin to elucidate these links and suggest that the UPR directly regulates lipid metabolism to coordinate a systemic response to ER stress inducing conditions.

## CELL NON‐AUTONOMOUS PARACRINE/ENDOCRINE SIGNALLING BY BIOACTIVE LIPIDS IN ER STRESS

Thus far we have observed associative evidence that the UPR can regulate the ceramide synthesis pathway to increase intracellular ceramide concentrations. However, recent studies have begun to elucidate the direct links between the UPR and the sphingolipid biosynthetic machinery. Moreover, for the ceramides to function as cell non‐autonomous signals these would need mechanisms for release or secretion from the cell of origin. As ceramides are hydrophobic these mechanisms would need to be an appropriate physiological process to overcome limitations to solubility and mediate extracellular transport.

Extracellular vesicles may play a key role in the secretion of ceramides from cells in which the UPR has been activated. Kakazu et al. identified that the UPR was induced in palmitate‐treated murine hepatocytes.^[48]^ Concomitantly, the intracellular concentration of ceramides was increased. The ceramides were packaged into extracellular vesicles and secreted from the hepatocytes.^[48]^ Production of the ceramides was directly linked to the UPR protein IRE1. Transgenic knockout of IRE1 in the hepatocytes inhibited the palmitate‐stimulated synthesis of ceramides and the release of the ceramide‐containing extracellular vesicles. Translationally, ceramide‐containing vesicles were increased in the circulation of patients with non‐alcoholic steatohepatitis; linking the secretion of ceramide‐enriched extracellular vesicles to liver disease in humans.

The interaction between the UPR and ceramide synthesis across multiple tissues, and the observation that these ceramides are released extracellularly, led us to hypothesise that lipid‐mediated paracrine and endocrine activation of the UPR may function as a mechanism of cell non‐autonomous communication of ER stress. Investigations in C2C12 immortalised murine myotubes and human primary myocytes exposed to palmitate exhibited increases in the expression of UPR genes including ATF4, heat shock protein family A (Hsp70) member 5 (Hspa5) and ER degradation enhancing alpha‐mannosidase like protein 1 (Edem1).^[11]^ Lipidomic profiling of palmitate‐treated myocyte media identified an increase in extracellular concentrations of long‐chain ceramides (ceramides C40:1 and C42:1). Media conditioned on palmitate‐treated myocytes exhibited the potential to induce the UPR and ER stress in naïve myocytes, suggesting the presence of a secretory ER stress‐inducing signal. Ceramides C40:1 and C42:1 were found to specifically induce UPR activation in myocytes and functioned additively when applied to the cells simultaneously. The long‐chain ceramides were enriched in the blood plasma and skeletal muscle of both mouse models of diabetes and human patients with T2DM and dyslipidaemia.^[11]^ Of the six CerS isoforms, CerS2 demonstrates a preference in the synthesis of long‐chain ceramides.^[49]^ Palmitate treatment of both mouse and human myocytes increased CerS2 expression. SiRNA‐mediated knockdown of CerS2 prevented the palmitate‐stimulated production of the long‐chain ceramides and their subsequent enrichment in the myocyte media.^[11]^ Media transferred from myocytes treated with palmitate and siRNA against CerS2 to naïve myocytes failed to induce the UPR. In a transgenic CerS2 catalytically‐inactive mouse model (CerS2 H/A mouse; two consecutive histidine for alanine substitutions in the CerS2 catalytic centre) long‐chain ceramide concentrations were decreased in both blood plasma and the skeletal muscle of the mice.^[11]^ In addition, skeletal muscle UPR gene expression was decreased in the CerS2 catalytically inactive mouse model. Therefore, CerS2‐generated long‐chain ceramides are cell non‐autonomous UPR‐inducing lipid signals.

Next, the synthesis of long‐chain ceramides in myocytes in response to lipotoxicity, through the direct regulation of specific UPR pathways, was investigated. Pharmacological inhibition of IRE1 had no effect on the increased intracellular and extracellular ceramide concentration of palmitate‐treated myocytes.^[11]^ However, both pharmacological inhibition and siRNA‐mediated knockdown of PERK in myocytes abrogated the palmitate‐induced synthesis and secretion of long‐chain ceramides.^[11]^ Thus, a role for the UPR kinase PERK in regulating myocyte sphingolipid synthesis in response to lipotoxicity was identified. However, the mechanisms through which PERK regulates CerS2 in the production of the long‐chain ceramide signals remains a key question to be determined.

As mentioned previously, palmitate treatment of hepatocytes activates secretion of ceramide‐rich extracellular vesicles.^[48]^ There is increasing recognition that bioactive lipid signals may be partitioned into extracellular vesicles.^[^ ^50^, ^51^ ^]^. Stimulation of extracellular vesicle release was also observed from palmitate‐treated myocytes.^[11]^ These extracellular vesicles were isolated and transferred to naïve myocytes in which they activated the UPR, reciprocally extracellular vesicle depleted media failed to induce the UPR in myocytes.^[11]^ These studies confirm that the extracellular vesicles contained the UPR‐inducing signal. Lipidomic analysis of the extracellular vesicles released from palmitate‐treated myocytes indicated that they were rich in long‐chain ceramides. Therefore, UPR‐induced extracellular vesicle‐mediated transport of ceramide from palmitate‐treated myocytes is required in the initiation of cell non‐autonomous UPR activation. In vivo, whether the UPR‐induced ceramide loaded EVs are targeted to specific tissues in the regulation of systemic ER stress, and the mechanisms through which this may be achieved, are yet to be established.

Finally, elements of the mechanism through which ceramides initiate ER stress and UPR activation in target tissues were elucidated. In myocytes treated with the long‐chain ceramides, lipidomic profiling identified that the intracellular concentration of their cognate dihydroceramides were increased.^[11]^ Dihydroceramides are ceramide precursors. This finding suggests exogenous ceramides may be imported and recycled within the cell. Independently, dihydroceramides have been found to drive activation of the UPR.^[52]^ Both pharmacological inhibition, and siRNA‐mediated knockdown of dihydroceramide desaturase 1 (Des1), the enzyme catalysing the conversion of dihydroceramides to ceramides, increased the dihydroceramide:ceramide ratio in human myocytes. Palmitate treatment in combination with Des1 knockdown synergistically enhanced UPR activation in the myotubes. A role for dihydroceramide accumulation in ER stress is observed in several cancers including gastric carcinoma and glioblastoma.^[^ ^53^, ^54^, ^55^ ^]^ This study takes this concept further by suggesting the conversion of extracellular ceramides to dihydroceramide has an emerging role in the cell non‐autonomous ceramide‐induced activation of UPR signalling. It also posits an interesting, and unanswered, question. What are the mechanisms controlling the metabolism of the ceramide signal back to dihydroceramides in target cells and then how do these dihydroceramides initiate ER stress and UPR activation? Answering these questions will pose significant challenges for future research.

## CONCLUSIONS

To conclude, we suggest that the intricate link between the UPR and sphingolipid and ceramide biosynthesis pathways, across multiple tissues, act as a communication node to couple the ER stress response to extracellular cell non‐autonomous bioactive lipid signals. These ceramide signals relay information on the status of the ER and its stress response in a paracrine and endocrine manner through extracellular vesicular transport. Whether UPR‐inducing stimuli, beyond the lipotoxic fatty acid species explored, also result in activation of this cell non‐autonomous bioactive lipid‐signalling pathway to produce systemic signals of ER stress remains an important point of clarification. It is possible to speculate that variety in the ER stress‐inducing stimuli may lead to modulation of UPR signalling and specificity in the downstream interorgan and non‐cell autonomous signals released by the stressed cell to regulate the systemic response to ER stress. This viewpoint would certainly be consistent with the variety of implicated signals (proteins, vesicular, bioactive lipids) in the studies discussed.

Into the future, we predict characterisation of the lipotoxicity‐mediated ER stress‐induced long‐chain ceramide signalling pathway for cell non‐autonomous activation of the UPR will occur across other key metabolic and ER stress‐sensitive tissues. The discovery that this process has a pathophysiological role in diseases including T2DM, obesity, dyslipidaemia and cancer highlights potential novel targets for therapeutic intervention in ER stress‐associated diseases.

## CONFLICT OF INTEREST STATEMENT

The authors declare they have no conflicts of interest.

## Data Availability

Data sharing is not applicable to this article as no new data were created or analysed in this study.
